# Serum Lipid, Amino Acid and Acylcarnitine Profiles of Obese Cats Supplemented with Dietary Choline and Fed to Maintenance Energy Requirements

**DOI:** 10.3390/ani11082196

**Published:** 2021-07-24

**Authors:** Adronie Verbrugghe, Alexandra Rankovic, Shafeeq Armstrong, Amanda Santarossa, Gordon M. Kirby, Marica Bakovic

**Affiliations:** 1 Department of Clinical Studies, Ontario Veterinary College, University of Guelph, 50 Stone Road East, Guelph, ON N1G 2W1, Canada; asantaro@uoguelph.ca; 2 Department of Biomedical Sciences, Ontario Veterinary College, University of Guelph, 50 Stone Road East, Guelph, ON N1G 2W1, Canada; arankovi@uoguelph.ca (A.R.); gkirby@uoguelph.ca (G.M.K.); 3 Department of Human Health and Nutritional Sciences, University of Guelph, 50 Stone Road East, Guelph, ON N1G 2W1, Canada; shafeeq.armstrong@gmail.com (S.A.); mbakovic@uoguelph.ca (M.B.)

**Keywords:** methyl donor, one carbon, obesity, methionine, fatty liver

## Abstract

**Simple Summary:**

Research has estimated that the majority of domestic cats are overweight or obese. Current weight-loss plans tend to have disappointing outcomes and are not without risk. During periods of severe energy restriction, obesity predisposes cats to developing fatty liver. Choline has been linked to fat metabolism in other animals but has not been studied in cats. Twelve obese cats were split into two groups and were fed a control diet (*n* = 6; 4587 mg choline/kg dry matter) or a high choline diet (*n* = 6; 18,957 mg choline/kg DM) for 5 weeks. Cats were fed to maintain body weight. Choline increased serum cholesterol, triacylglycerides, lipoproteins, and plasma methionine. It also decreased serum blood urea nitrogen and alkaline phosphatase as well as the ratio of plasma acylcarnitine to free carnitine. The results suggest that choline supplementation may increase fat transport out of the liver and help maintain liver health in obese cats. Choline supplementation may prove useful for safe weight loss in obese cats by minimizing the risks of fatty liver.

**Abstract:**

Obesity is a health concern for domestic cats. Obesity and severe energy restriction predispose cats to feline hepatic lipidosis. As choline is linked to lipid metabolism, we hypothesized that dietary choline supplementation would assist in reducing hepatic fat through increased lipoprotein transport and fatty acid oxidation. Twelve obese cats (body condition score [BCS] ≥ 8/9) were split into two groups. Cats were fed a control (*n* = 6; 4587 mg choline/kg dry matter [DM]) or a high choline diet (*n* = 6; 18,957 mg choline/kg DM) for 5 weeks, for adult maintenance. On days 0 and 35, fasted blood was collected, and the body composition was assessed. Serum lipoprotein and biochemistry profiles, plasma amino acids and plasma acylcarnitines were analyzed. The body weight, BCS and body composition were unaffected (*p* > 0.05). Choline increased the serum cholesterol, triacylglycerides, high-density lipoprotein cholesterol, low-density lipoprotein cholesterol, very low-density lipoprotein cholesterol and plasma methionine (*p* < 0.05) and decreased the serum blood urea nitrogen and alkaline phosphatase (*p* < 0.05). Choline also reduced the plasma acylcarnitine to free carnitine ratio (*p* = 0.006). Choline may assist in eliminating hepatic fat through increased fat mobilization and enhanced methionine recycling.

## 1. Introduction

Akin to the obesity epidemic in humans, obesity is largely prevalent in domestic cats. Globally, it is estimated that 19–63% of cats are obese, depending on the country and the criteria used for classification [1,2,3,4,5,6,7,8,9,10,11]. This high number of obese animals is of concern, as obesity has the potential to lead to damaging health conditions including osteoarthritis, lower urinary tract diseases and diabetes mellitus [1,3,12]; subsequently resulting in a reduced quality of life [13].

Specifically, obese cats were found to be five times more likely to develop issues with mobility and four times more likely to develop diabetes mellitus, as compared with lean cats (with an optimal body condition) [1]. Numerous risk factors have been implicated with weight gain and obesity in cats. These include environment-specific factors, such as indoor housing, lack of physical exercise and ad libitum feeding [3,7,8]. Animal-specific factors, including age, sex and gonadectomy, have also been implicated as risk factors for the development of obesity [1,3,4,7,9,10,14,15].

Although weight reduction is recommended for cats that are overweight or obese, weight-loss attempts can be disappointing and can also be associated with health risks [16,17,18]. A weekly weight loss at a rate of 0.5–2% of the initial body weight is clinically considered safe [19,20]. Rapid weight loss that exceeds this recommendation predisposes cats to feline hepatic lipidosis (FHL); defined as an accumulation of lipids within the liver [18]. Feline hepatic lipidosis is a common form of liver disease, affecting an estimated 0.16% of cats in North America [21].

It is most commonly diagnosed in cats that were initially overweight or obese [22]. Although the pathophysiology of FHL is not fully understood, reduced energy intake appears to be the primary factor in its development [23]. It is reported that FHL can occur within 2 days to 2 weeks in a clinical setting, depending on how overweight or obese the animal initially was, in combination with the degree of energy restriction imposed [22,24]. An estimated restriction of 50–75% of a cat’s maintenance energy requirements is required in order to induce FHL [25,26,27]. When left untreated, FHL commonly leads to liver failure and/or death in affected cats [28].

Choline is an essential nutrient that gives rise to numerous important products within the body, including phosphatidylcholine (PC), acetylcholine and betaine [29,30]. By doing so, choline plays a critical role in numerous pathways, including neurotransmission and cell signalling. However, of interest to the topic of FHL and feline obesity, are the roles that choline plays in lipid metabolism and hepatic function. Choline has been metabolically linked to lipid metabolism in other animal models. In livestock, such as poultry and swine, the supplementation of choline or its derivative betaine, has been shown to improve lean carcass composition and decrease the deposition of fat [31,32,33,34,35,36,37].

Moreover, supplementation of choline was shown to reduce the accumulation of hepatic fat in obese mice, through increased lipolysis and mobilization of fat outside of the liver [38]. Choline assists in the removal of fat from the liver through synthesis of PC, which is a structural component of very low-density lipoproteins (VLDL) [39]. Moreover, the choline oxidation product, betaine, stimulates homocysteine re-methylation to methionine [40]. By doing so, S-adenosyl-methionine (SAM) is produced, resulting in increases in both PC and carnitine [41]. Carnitine is essential for the mobilization of long-chain fatty acids to the inner membrane of the mitochondria for oxidation [42].

With this in mind, we hypothesized that feeding supplemental choline to obese cats could assist in the elimination of hepatic fat through increased lipoprotein transport and enhanced fatty acid oxidation within the liver. The purpose of this study was to assess the effects of dietary choline supplementation in obese cats fed at maintenance energy requirement on serum lipid, amino acid and acylcarnitine profile. Emphasis was placed on assessing body composition, serum lipid profile, as well as plasma amino acid and acylcarnitine profiles.

## 2. Materials and Methods

All experimental procedures were approved by the University of Guelph Animal Care Committee (AUP#2494). All procedures were in accordance with national and institutional guidelines for the care and use of animals.

### 2.1. Animals and Housing

Twelve domestic shorthair cats (six neutered males and six spayed females) were included in this study. All cats were considered chronically obese, with a body condition score (BCS) of ≥8/9 [43]. At the start of the study, the cats had a mean body weight (BW) of 7.4 ± 0.3 kg (mean ± SEM; range: 5.9–8.9 kg) and a mean age of 9.0 ± 0.7 years (mean ± SEM; range: 5–13 years). Although chronically obese, all cats were healthy based on a physical exam, complete blood count (CBC), and serum biochemical analysis. The cats were housed at a private contract research facility. Throughout the study cats were kept in their usual group housing. The cats were separated from 4 PM to 7 AM daily, in order to be fed individually. Water was available ad libitum all day for each cat.

### 2.2. Experimental Diets

The two experimental diets (Elmira Pet Products, Elmira, ON, Canada) used for this research were non-commercial extruded cat foods formulated according to the feline adult nutrient profile of the American Association of Feed Control Officials (AAFCO). Choline chloride (99% choline chloride, Vitacholine, Balchem, New Hampton, NY, USA) was used as the dietary choline supplement. Choline chloride was added into the diets prior to extrusion at 0.19% for the control diet and 2.86% for the high choline diet, aiming at 300 mg per 100 g dry matter (DM) and 2679 mg per 100 g DM based on formulation, respectively. Dietary analysis showed that the control diet contained 459 mg choline per 100 g DM, compared to the high choline diet, which contained 1896 mg per 100 g DM. Both diets had the same ingredient and nutrient profile (Table 1), apart from the additional choline chloride supplementation in the high choline diet.

The diets were analysed for moisture, protein, crude fat and ash in-house by Elmira Pet Products (Elmira ON, Canada), according to the Association of Official Analytical Chemists (AOAC). Moisture was assessed by gravimetric analysis (AOAC 935.29), crude protein by combustion (AOAC 990.03), crude fat by acid hydrolysis (AOAC 954.02) and ash by gravimetric analysis (AOAC 942.05) [44]. Crude fiber was analysed by SGS Canada Inc. (Guelph, ON, Canada), in accordance with the American Oil Chemists Society (AOCS), using the filter bag technique (AOCS Ba6a-05) [45]. Metabolizable energy was estimated using predictive equations, according to a four-step calculation based on the calculation of gross energy (GE) and digestible energy (DE) [19].

The choline, cobalamine, pyridoxine and folic acid analyses were performed by Maxxam Analytics (Mississauga, ON, Canada). Choline was assessed by the enzymatic colorimetric method (AOAC 999.14), cobalamine by the turbidimetric method (AOAC 986.23), pyridoxine by the microbiological method (AOAC 985.32 (modified)) and folic acid by the triple enzyme microbiological method (AOAC 2004.5) [44]. The dietary free amino acid (methionine, lysine and threonine) and total amino acid contents of each diet were analysed by AminoLab^®^, Evonik Industries (Kennesaw, GA, USA) using high-performance liquid chromatography (HPLC) post-column derivatization (AOAC 999.13) and performic acid oxidation (AOAC 994.12), respectively.

### 2.3. Experimental Design

Prior to the treatment period, a 4-week adaptation period was implemented in order for the accurate determination of each cat’s individual maintenance energy requirements. All cats were fed the control diet during the adaptation period. Initially, the amount of food offered was calculated based on the maintenance energy requirement for obese cats according to the National Research Council (NRC) (130 kcal/kg BW^0.4^) [19]. Leftovers from each meal were weighed daily and used to calculate the daily food intake for each cat. The cats were weighed twice weekly, and the BCS was assessed on a nine-point-scale and recorded weekly [43]. The amount of food offered was adjusted to maintain a constant BW and BCS.

At the end of the adaptation period, the cats were divided into two groups, balanced for gender, BW and age. During the 5-week treatment period, one group of cats (*n* = 6) continued to receive the control diet, while the other group (*n* = 6) was fed the high choline diet. Cats continued to receive the same amount of food that was offered at the end of the adaption period and were shown to maintain a stable BW and BCS. Recording of the food intake, BW and BCS followed the same schedule as during the adaptation period.

### 2.4. Blood Collection and Analyses

Blood samples (16 mL) were taken from the jugular vein after a 12-h fast on day 0 and day 35 of the treatment period. Whole blood (10 mL) was collected in plastic serum tubes (Plus Plastic Serum Tubes, Vacutainer^®^, Becton Dickinson, Franklin Lakes, NJ, USA) for analyses of serum cholesterol (CHOL), high-density lipoprotein cholesterol (HDL-C), triacylglycerides (TAG), non-esterified fatty acids (NEFA), alkaline phosphatase (ALP), alanine aminotransferase (ALT), blood urea nitrogen (BUN), creatinine (CREAT), glucose, insulin and leptin.

Serum was obtained after the centrifugation of whole blood at 2500× *g* for 15 min at 4 °C. Serum was stored at −20 °C until analyzed. Whole blood (6 mL) was also collected in EDTA tubes (Spray-coated K2EDTA Tubes, Vacutainer^®^, Becton Dickinson, Franklin Lakes, NJ, USA) and stored as dried blood spots (375 μL) using Whatman^TM^ 903^TM^ Protein Saver Cards (GE Healthcare Bio-Sciences Corp. Westborough, MA, USA) at −20 °C until the amino acid and acylcarnitine analyses.

The serum CHOL, TAG, NEFA, HDL-C, ALP, ALT, BUN, CREAT and glucose concentrations were analysed photometrically at the Animal Health Laboratory, University of Guelph (Guelph, ON, Canada), using a Roche Cobas 6000 c501 Analyzer (Roche Diagnostics, Basel, Switzerland). Very low-density lipoprotein cholesterol (VLDL-C) and low-density lipoprotein cholesterol (LDL-C) were calculated using the Friedewald equation (VLDL (mmol/L) = TAG (mmol/L)/2.2; LDL-C (mmol/L) = total CHOL (mmol/L) − HDL-C (mmol/L) − VLDL (mmol/L)). This equation has been established for use in humans and has previously been used in cats [46,47,48].

Serum insulin concentrations were determined by use of a commercially available ELISA kit (Feline Insulin ELISA, Mercodia AB, Uppsala, Sweden), validated for use in cats [49]. The Bennet Index (BI), the homeostasis model assessment (HOMA), insulin to glucose ratio (I-G) and the quantitative insulin sensitivity check index (QUICKI) were calculated as previously described by Appleton et al. (2005) [50]. Serum leptin concentrations were determined at the Laboratory of Livestock Physiology, Immunology and Genetics, Department of Biosystems, K.U. Leuven (Leuven, Belgium) using a commercially available RIA kit (Multi-species Leptin RIA kit, Linco Research Inc., St Charles, MO, USA), validated for use in cats [51].

Quantitative electrospray tandem mass spectrometry was performed at the Department of Clinical Chemistry, Laboratory of Metabolic Disorders, University Hospital Ghent (Ghent, Belgium) to determine the plasma free and total carnitine, acylcarnitine and amino acid profile as described by Rizzo et al. (2003) and Vreken et al. (2002) [52,53]. Short-, medium- and long-chain acylcarnitines were calculated as derivates with carbon chains C2–C5, C6–C12 and C14–C18, respectively. The ratio of plasma acylcarnitines to free carnitine (AC/FC) was calculated by dividing the total concentration of acylcarnitines by the free carnitine concentration.

### 2.5. Dual Energy X-ray Absorptiometry

The body composition was assessed by dual energy X-ray absorptiometry (DEXA), using a commercially available machine (QDR-4500 Acclaim Series Elite, Hologic Inc, Bedford, MA, USA). The cats were positioned in ventral recumbency on the scanner table for all scans [54]. Scans were performed once at each time point (days 0 and 35) for each cat. Cats were anesthetized by propofol induction at 6.5 mg/kg BW intravenously. Standard monitoring of vital signs was applied throughout anaesthesia. The area, bone mineral content (BMC), bone mineral density (BMD), fat mass (FM), lean body mass (LBM), total mass (TM) and body fat percentage (BF%) were analyzed and calculated from the resulting images of the scans using a commercially available software (Apex, Version 2.3, Hologic Inc, Bedford, MA, USA).

### 2.6. Statistical Analyses

Statistical analysis was performed using SPSS^®^ Statistics (Version 26, IBM^®^, New York, NY, USA). Prior to analysis, the data were tested for normality using the Shapiro–Wilk test. For normally distributed data, a repeated measures analysis of variance (ANOVA) model was used, with time as the within subject factor and diet as the between subject factor. The time, diet and the time × diet interaction were assessed. When any significance occurred, a Bonferroni post-hoc test was performed to assess multiple comparisons for the fixed effects (time and diet) and their respective interaction. Data that was not normally distributed (BCS), was log transformed prior to analysis. Statistical significance was set at *p* < 0.05. The results are expressed as the mean ± SEM.

## 3. Results

### 3.1. Energy, Food and Choline Intake

All cats ate their assigned diet and showed no signs of illness and/or maldigestion. The mean energy intake was similar during the treatment period as during the adaptation period and was also similar between diet groups (*P*_time_ = 0.549; *P*_time × diet_ = 0.994; and *P*_diet_ = 0.442). Accordingly, the food intake did not differ between diet groups or between the testing and adaptation periods (*P*_time_ = 0.588; *P*_time × diet_ = 0.987; and *P*_diet_ = 0.093).

Cats on the control diet consumed an average of 283.82 ± 9.41 mg choline per day during the treatment period (77.27 ± 4.60 mg choline/kg BW^0.67^; and 40.85 ± 3.13 mg choline/kg BW). In comparison, the group on the high-choline diet consumed 1252.29 ± 34.23 mg choline per day (321.98 ± 5.71 mg choline/kg BW^0.67^; and 165.24 ± 4.85 mg choline/kg BW).

### 3.2. Body Weight, Body Condition Score and Body Composition

There were no changes in the weekly BCS between the two diet groups throughout the trial (*P*_time_ = 0.343; *P*_time × diet_ = 0.453; and *P*_diet_ = 0.387). Additionally, there were no significant effects of time, diet or time x diet interaction for BW or body composition; including area, BMC, BMD, FM, LBM, TM and BF% (*p* > 0.05; Table 2).

### 3.3. Serum Lipid Profile

**Figure 1:**
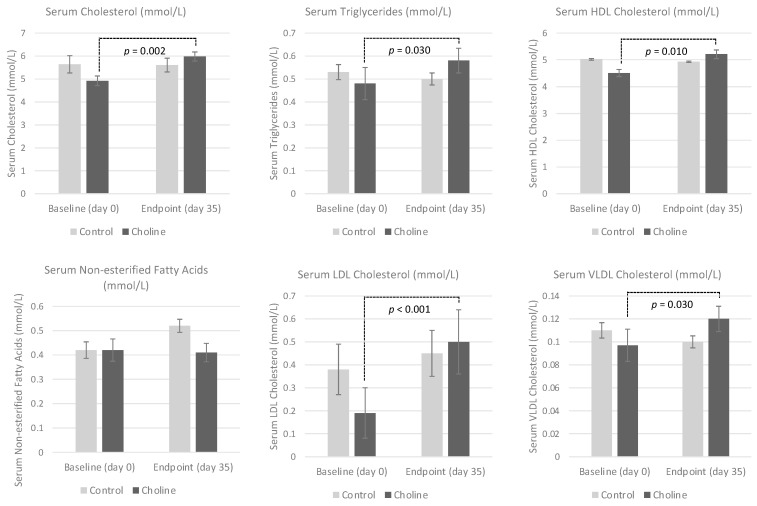
Serum lipoprotein concentrations (mmol/L) in 12 chronically obese cats following 5 weeks of a control extruded cat food (458.7 mg choline/100 g dry matter (DM)) (*n* = 6) or a high-choline extruded cat food (1895.7 mg choline/100 g DM) (*n* = 6). Both groups were fed at maintenance energy requirements. Values are expressed as the mean ± SEM.

There was a significant time × diet interaction for serum TAG, HDL-C and VLDL (*P*_time_ _× diet_ = 0.038, *P*_time_ _× diet_ = 0.029 and *P*_time_ _× diet_ = 0.038, respectively), as the serum concentrations increased in the cats consuming the high choline diet after 5 weeks (Table 3; Figure 1). However, individual effects of diet or time were not observed for these serum parameters (*p* > 0.05). Serum CHOL and LDL-C were affected by time (*P*_time_ = 0.017 and *P*_time_ = 0.001, respectively), with significant increases being noted from baseline to endpoint in the high choline group (*P*_time_ _× diet_ = 0.013 and *P*_time_ _× diet_ = 0.014, respectively). An individual effect of diet on serum CHOL and LDL-C did not occur (*P*_diet_ = 0.634 and *P*_diet_ = 0.683, respectively). There were no changes in the serum NEFA between groups over the 5-week treatment period (*P*_time_ = 0.110, *P*_time_ _× diet_ = 0.279 and *P*_diet_ = 0.090).

### 3.4. Serum Liver Enzymes

Although there was no individual effect of diet on the serum ALP (*P*_Diet_ = 0.712), the concentration of ALP decreased for both groups over the 5-week treatment period (*P*_time_ = 0.012). The decrease in serum ALP from baseline to endpoint was significant for the cats on the high choline group (*P*_time_ _× diet_ = 0. 027). There were no changes in the serum ALT concentrations over time and between groups (*P*_time_ = 0.087, *P*_time_ _× diet_ = 0.319 and *P*_diet_ = 0.879).

### 3.5. Serum Creatinine and Blood Urea Nitrogen

Concentrations of the serum BUN were lower at the endpoint compared to the baseline in both groups (*P*_time_ = 0.027). However, there were no significant effects of diet or time × diet interaction (*P*_diet_ = 0.470 and *P*_time_ _× diet_ = 0.099). The serum CREAT concentrations were not affected by diet and time (*P*_time_ = 0.802, *P*_diet_ = 0.509 and *P*_time_ _× diet_ = 0.643).

### 3.6. Serum Glucose, Insulin and Leptin

Serum glucose concentrations tended to decrease after 5 weeks in the high choline group but not in the control group (*P*_time_ _× diet_ = 0.051). There were no individual effects of time or diet on glucose (*P*_time_ = 0.747 and *P*_diet_ = 0.467). There were no effects of time, diet or time x diet interaction for serum insulin, I-G, BI, HOMA or QUICKI Index (*p* > 0.05). An individual effect of time was present for serum leptin (*P*_time_ = 0.039), as concentrations decreased for both groups over the 5-week treatment period, yet the effects of diet and time x diet interaction were not significant (*P*_diet_ = 0.377 and *P*_time_ _× diet_ = 0.224).

### 3.7. Plasma Amino Acid Profile

Of the plasma amino acids analyzed (glycine, alanine, valine, leucine, ornithine, methionine, phenylalanine, citrulline and tyrosine), significant changes were only found for methionine (*P*_time_ = 0.010; *P*_diet_ = 0.190; and *P*_time_ _× diet_ = 0.005; data not shown). The plasma methionine concentrations (µmol/L) increased from the beginning to the end of the treatment period in the high choline group (baseline: 71.63 ± 3.26; endpoint: 98.20 ± 5.78); this change did not occur in the control group (baseline: 78.49 ± 3.26; endpoint: 76.84 ± 5.47). No diet differences occurred at baseline, yet the plasma methionine concentration at the end of the treatment period was significantly greater for the high choline group as compared to the control group.

### 3.8. Plasma Acylcarnitine Profile

The plasma free carnitine, total carnitine and total acylcarnitine concentrations were not affected by diet, time or time × diet interaction (*p* > 0.05; data not shown). An effect of diet was present for AC/FC ratio (*P*_diet_ = 0.006). The AC/FC ratio at endpoint was significantly lower in the high choline group compared to the control group. No effect of time or diet–time interaction was present (*P*_time_ = 0.296 and *P*_time_ _× diet_ = 0.811, respectively). No effects of diet, time or time × diet interaction were seen in combined plasma short-, medium- or long chained acylcarnitine concentrations (*p* > 0.05). An effect of time was present for both malonylcarnitine (C3-DC) and octanoylcarnitine (C8) (*P*_time_ = 0.050 and *P*_time_ = 0.015, respectively).

The plasma concentrations of these acylcarnitines decreased in both diet groups after 5 weeks. However, the plasma concentrations of C3-DC and C8 was not affected by diet (*P*_diet_ = 0.465 and *P*_diet_ = 0.200, respectively) or time × diet interaction (*P*_time_ _× diet_ = 0.245 and *P*_time_ _× diet_ = 0.603, respectively). Methylmalonylcarnitine (C4-DC), palmitoleic acid (C16:1) and 3OH-palmitoylcarnitine (3OH-C16) were affected only by diet (*P*_diet_ = 0.002, *P*_diet_ ≤ 0.001 and *P*_diet_ = 0.009, respectively). Plasma concentrations of the aforementioned acylcarnitines were lower at both baseline and endpoint in the high choline group as compared to the control group. However, the effects of time (*P*_time_ = 0.821, *P*_time_ = 0.416 and *P*_time_ = 0.267, respectively) and time x diet interaction (*P*_time_ _× diet_ = 0.134, *P*_time_ _× diet_ = 0.212 and *P*_time_ _× diet_ = 0.148, respectively) were insignificant.

A significant time x diet interaction was present for valerylcarnitine (C5) and 3OH-dodecanoylcarnitine (3OH-C12) (*P*_time_ _× diet_ = 0.035 and *P*_time_ _× diet_ = 0.040, respectively). Plasma concentrations of C5 decreased from baseline to endpoint in the control group but conversely increased in the high choline group. However, there were no individual effects of time (*P*_time_ = 0.713) or diet (*P*_diet_ = 0.311). In comparison, plasma 3OH-C12 concentrations increased after 5 weeks for the control group but remained consistent for the high choline group. Similarly, there were no effects of time (*P*_time_ = 0.097) or diet (*P*_diet_ = 0.209) on plasma 3OH-C12.

## 4. Discussion

To the authors’ knowledge, the current study is the first to investigate the effects of choline supplementation on serum lipid, amino acid and acylcarnitine profile in obese cats. Choline is considered an essential nutrient [55,56], and, when de novo synthesis in the liver is inadequate, small concentrations of dietary choline can prevent and/or resolve numerous health conditions in both animals and humans, including fatty liver [57]. As such, researchers have proposed that choline may be involved with the pathogenesis and treatment of FHL [26,58]. Choline has important roles for numerous metabolic pathways within the body, including being a precursor for the synthesis of PC and being an important source of methyl group donor betaine [29,30,55]. As such, modifying the intake of choline can alter lipid and methionine metabolism.

Phosphatidylcholine is essential for the structural integrity of the plasma membrane and is integral for the lipid metabolism. Approximately 95% of the choline in tissue is found as PC [59]. In relation to the lipid metabolism, PC is a necessary component of VLDL [39]. This is especially important to consider for liver health, as fatty acids are incorporated into TAG, which accumulate in the liver. Under normal circumstances, the TAG will be packed with PC into VLDL, and secreted out of the hepatocytes and into circulation. As a result, choline deficiency can diminish synthesis of PC, leading to increased TAG accumulation within the liver [60,61,62,63,64].

**Figure 2:**
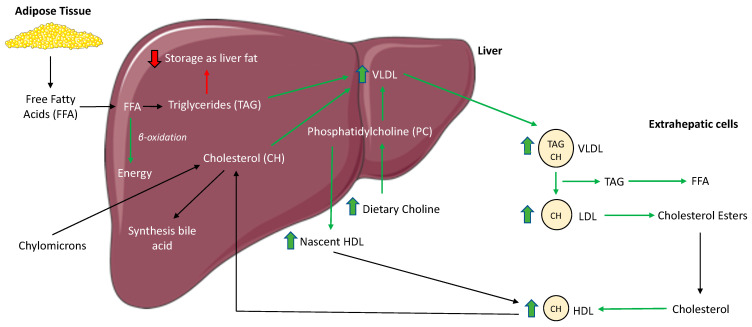
The proposed mechanism by which supplemental dietary choline alters the hepatic lipid and lipoprotein metabolism in obese cats. An increase in dietary choline allows for an increase in phosphatidylcholine (PC), necessary for the formation of very-low-density lipoproteins (VLDL). Hepatic triacylglycerides (TAG) and cholesterol are packed into VLDL and put into circulation for distribution within the body. The formation of nascent high-density lipoprotein (HDL) within the liver is also dependent on PC. PC: phosphatidylcholine; VLDL: very-low-density lipoprotein; HDL: high-density lipoprotein; LDL: low-density lipoprotein; TAG: triacylglycerides; FFA: free fatty acids.

Obese cats have increased accumulation of hepatic TAG compared to lean cats [65]. Although liver tissue samples were not collected in the present study, increased serum TAG and VLDL were observed with dietary choline supplementation, suggesting increased export of TAG and VLDL from the liver into circulation (Figure 2). The concentrations of serum CHOL, HDL-C and LDL-C also increased after 5 weeks in cats receiving the high choline diet.

These are unsurprising findings due to the increase in VLDL, the role that HDL-C and LDL-C have in transporting cholesterol and the similar involvement of PC in the assembly of these lipoproteins [66,67]. The risks of cardiovascular disease in regards to increased serum lipids in cats are believed to be minimal, as dyslipidemia on its own does not appear to induce atherosclerosis or hypertension [68,69] as it might in other species [70].

The results of the present study align with previous findings in rats, which demonstrated that rats fed choline deficient diets had significantly lower levels of serum HDL-C, LDL-C, VLDL, TAG and CHOL as compared to those receiving choline [60,61,71,72]. Additionally, young lambs fed rumen-protected choline similarly had increased concentrations of serum HDL-C and LDL-C compared to the control group fed a diet with no rumen-protected choline [73]. Lien et al. (1998) also found that ducks consuming supplemental choline during the growing period had significantly increased serum VLDL concentrations as compared to the control. Ducks consuming higher doses during the laying period had significantly greater serum concentrations of both VLDL and HDL-C as compared to the control group [74].

In the present study, a decrease in serum ALP was observed with additional choline supplementation, which aligns with previous research in humans, dairy cows and piglets [75,76,77,78]. This decrease in serum ALP suggests that additional choline supplementation improves the hepatic health and function in these obese cats, as increased ALP concentrations have previously been associated with numerous hepatobiliary diseases in cats, including FHL and bile duct occlusion [22,79,80]. However, as biopsies and ultrasonography were not performed in the present study, hepatic health could not be assessed in these cats, aside from a lack of a hepatic enzymopathy.

Choline also participates in the one-carbon cycle through its derivative betaine (trimethylglycine) [81]. In the liver, choline is first oxidized by choline dehydrogenase to betaine aldehyde [82]. Following this, in the presence of NAD +, betaine aldehyde is oxidized to betaine by betaine aldehyde dehydrogenase in the mitochondria [83]. Betaine is a methyl group donor in the one-carbon cycle, as it can re-methylate homocysteine to methionine through a reaction catalyzed by betaine-homocysteine-methyltransferase (BHMT) [40].

In the present study, the supplementation of additional choline resulted in higher levels of plasma methionine, likely through increased concentrations of betaine and increased re-methylation of homocysteine. Supplementation with additional dietary choline also tended to reduce the serum BUN and glucose. This could suggest increased recycling of methionine and potentially increased protein synthesis as opposed to amino acid degradation, thus, resulting in gluconeogenesis and the formation of urea [84].

The re-methylation of homocysteine through BHMT not only converts methionine and detoxifies homocysteine but also results in the production of S-adenosylmethionine (SAM) [85]. Research in mice showed that, when fed a choline-deficient diet, the concentrations of hepatic SAM were decreased by up to half in these animals [86,87,88,89]. The generation of SAM is important as it is the main methyl group donor in numerous metabolic pathways and is involved in the synthesis of proteins, hormones and phospholipids as well as in DNA methylation [90].

More specifically, SAM is an important methyl donor for the methylation and synthesis of carnitine [41]. The entry of fatty acids into the mitochondria for beta oxidation relies on carnitine for transport of the fatty acids from circulation [42]. The oxidation of fatty acids in the mitochondria is important as it is the main pathway for disposal of fatty acids under normal physiological conditions. The oxidation of fatty acids is also important as it results in the production of acetyl-CoA by the Krebs cycle, allowing for the production of ATP by ATP synthase [91].

Acylcarnitines are products of fatty acids entering the mitochondria for beta oxidation [92]. Previous research in mice found that the supplementation of methyl donors reduced plasma acylcarnitine concentrations through regulation of gene expression related to lipogenesis, lipolysis and fatty acid oxidation [38,93].A decrease in plasma acylcarnitines may indicate that mitochondrial oxidation of fatty acids improved [93]. Increased plasma acylcarnitine concentrations reflect incomplete beta oxidation of fatty acids and have been associated with insulin resistance [92,94].

An increased concentrations of plasma acylcarnitines is commonly observed in conjunction with obesity [92,95,96]. In the present study, choline supplementation did not significantly alter the plasma acylcarnitine concentrations. However, a reduced ratio of AC/FC was observed with choline supplementation in obese cats, suggesting improvements in fatty acid utilization, which may contribute to lowering liver fat accumulation.

Methylmalonyl carnitine (C4-DC), palmitoleic acid (C16:1) and 3OH-palmitoylcarnitine (3OH-C16) were lower in the high choline group at the baseline and endpoint compared to the control group, which may be attributed to individual variations in the cats within these groups. However, it is unclear why malonylcarnitine (C3-DC) and octanoylcarnitine (C8) decreased in both groups throughout the trial. Valerylcarnitine (C5) decreased from the baseline to endpoint in the control group but increased in the high choline group.

Valerylcarnitine is a by-product of the branched chain amino acid (BCAA) catabolism. Previous research by Sivanesan et al. (2018) suggested upregulation of the amino acid catabolism in mice supplemented additional choline. Specifically, BCAA concentrations (isoleucine and valine) were reduced with supplementation in the mice [97]. However, there were no significant changes in the BCAA analyzed in the present study (leucine and valine). An increase in the BCAA catabolism is suggested to be associated with insulin resistance and obesity.

Previous research has found that C5 is elevated in humans suffering from insulin resistance, obesity and/or steatosis [96,98]. However, to the authors’ knowledge, these relationships have not been established in cats. An increase in 3OH-dodecanoylcarnitine (3OH-C12) occurred in the control group after 5 weeks, while concentrations remained consistent in the high choline group. Dodecanoylcarnitine was previously implicated as a marker of obesity-induced inflammation in rats fed nutrient poor “cafeteria diets” [99].

In the present study, cats receiving the control and high choline diets consumed an average of 284 mg and 1252 mg of choline, respectively, per day. Although some endogenous biosynthesis of choline does occur through the breakdown of PC [41], the NRC recommends that adults cats receive a minimum of 50 mg/kg BW^0.67^ choline daily. The NRC recommended allowance (RA) is 63 mg/kg BW^0.67^ daily [19]. In the present study, the cats on the high choline diet consumed more than five times the NRC RA and close to seven times the NRC minimum requirement daily.

In humans, choline deficiency can lead to numerous health conditions, including the development of non-alcoholic fatty liver and dysfunctions in muscle function [75,100,101,102]. Due to a lack of information investigating the choline requirements of cats, the minimum allowance established by the NRC is based on research by Schaeffer et al. (1982) investigating growth and hepatic lipid accumulation in growing kittens with choline supplementation [103]. It is unknown whether this level of choline can be considered optimal for health in adult cats, especially in overweight cats fed at maintenance energy requirement or during energy restriction and weight loss [104].

Research in cats has found that choline is often the limiting nutrient, provided below NRC’s minimum requirement and/or RA for adult maintenance. A study observed that all obese cats undergoing weight loss and consuming a specific commercially available veterinary therapeutic weight loss diet (350 mg choline/100 g diet as fed) had a daily choline intake that was less than the NRC RA [105]. The daily choline intake of the majority of these cats was also below the NRC minimum requirement.

This finding is especially alarming due to the individual risks that both weight loss in cats and choline deficiency pose on hepatic health and function. Additionally, choline was the nutrient most frequently provided below NRC RA in feline homemade diet formulations intended to be complete and balanced for adult maintenance [106]. Choline was less than NRC RA in almost 90% of the homemade recipes evaluated in said study.

Currently there is evidence that providing additional dietary choline, on top of an animal’s minimum requirement can enhance the lipid metabolism [31,32,33,34,35,36,37]. There were no changes in the BW, BCS or body composition observed between treatment groups in the present study. Additionally, there were no changes in the leptin concentration with additional choline supplementation. However, given the limited duration of testing and that the cats were fed at maintenance energy requirement, we did not expect to see significant decreases in these measures. Another possible limitation of the present study may have been its limited sample size, although similar sample sizes have previously been published in rodent research [38].

As cats in the high choline group consumed five-times the NRC RA, future studies should focus on identifying the minimum dose of choline necessary stimulate hepatic fat mobilization in overweight and obese cats. Furthermore, evaluation of the liver size by ultrasonography and biopsies of the liver should be performed in order to confidently assess the liver histology and expression of genes related to the hepatic energy metabolism, including gluconeogenesis, lipogenesis, lipolysis and VLDL secretion. Moving forward it will also be important to pair the results of this research with the already existing knowledge of weight loss.

It is known that cats are more susceptible to FHL. Although the precise pathogenesis remains a mystery, most researchers believe that multiple factors associated with the unique pathways of protein and lipid metabolism in cats are involved. Obesity, energy restriction and essential nutrient deficiency have been put forward as major risk factors for FHL [23,107]. The degree of energy restriction needed to induce hepatic lipidosis was identified to be between 50% and 75% [25,26,27]. Caloric restriction paired with supplemental dietary choline could potentially offer an additive, beneficial effect with respect to weight loss and a decrease in hepatic lipids.

## 5. Conclusions

In conclusion, the supplementation of additional choline on top of the NRC requirements for adult maintenance could assist in eliminating hepatic fat through increased lipid mobilization, enhanced methionine recycling and fatty acid oxidation in obese cats fed at maintenance energy requirements. Further research is warranted to further elucidate the metabolic effects of dietary choline and its benefits for obesity prevention and weight loss in cats.

## Figures and Tables

**Table 1:** Proximate analysis and dietary analyses of chloride, sodium, selected B-vitamins and amino acids of two experimental diets: a control diet and a high choline diet (DMB).

		Control Diet	High Choline Diet
Moisture	% as fed	5.60	5.05
Protein	% DM	35.53	36.70
Fat	% DM	19.81	16.75
Ash	% DM	6.43	6.47
Crude Fibre	% DM	2.75	3.58
NFE ^1^	% DM	35.48	36.50
ME ^2^	kcal/100g	414.31	400.43
Chloride	% DM	0.77	1.33
Sodium	% DM	0.55	0.59
Choline	mg/100 g	458.69	1895.73
Cobalamine (B12)	μg/100g	8.47	10.53
Folate (B9)	mg/100g	0.32	0.42
Pyridoxine (B6)	mg/100g	1.02	1.37
Methionine	% DM	0.94	0.94
Cysteine	% DM	0.48	0.47

DMB: dry matter basis; NFE: nitrogen free extract; ME: metabolizable energy Values reported on DMB, except for moisture; ^1^ NFE (%) = 100 – moisture − protein – fat – fibre − ash; ^2^ Estimated by a four-step calculation [19]. Ingredients: chicken meal, brewer’s rice, corn gluten meal, whole corn, chicken, poultry fat, whole oat flour, yellow pea hulls, whole dried egg, dried chicken, beet pulp, salmon oil, potassium chloride, sodium chloride, choline chloride, DL-methionine, taurine, vitamin and mineral premix, and yucca extract.

**Table 2:** Body weight and body composition in 12 chronically obese cats at baseline and after feeding a control food (458.7 mg choline/100g DM) (*n* = 6) or a high-choline food (1895.7 mg choline/100g DM) (*n* = 6) for 5 weeks.

	Control Diet		High Choline Diet		*p*-Value		
	Baseline	5 Weeks	Baseline	5 Weeks	Time	Diet	T × D
BCS	8.7 ± 0.2	8.5 ± 0.2	9.0 ± 0.2	8.8 ± 0.2	0.343	0.387	0.453
BW/kg	7.1 ± 0.5	7.1 ± 0.5	7.6 ± 0.4	7.6 ± 0.3	0.915	0.491	0.409
DEXA SCAN							
Area/cm^2^	578.3 ± 28.5	574.5 ± 23.1	600.8 ± 20.7	586.5 ± 19.2	0.075	0.607	0.272
BMC/g	167.7 ± 12.4	167.6 ± 11.2	167.4 ± 6.6	164.6 ± 6.4	0.180	0.901	0.191
BMD g/cm^2^	167.7 ± 12.4	167.6 ± 11.2	167.4 ± 6.6	164.6 ± 6.4	0.180	0.901	0.191
FM/g	2784.8 ± 295.5	2919.5 ± 283.5	3034.7 ± 292.6	2995.0 ± 357.4	0.497	0.714	0.225
LBM/g	4427.2 ± 327.7	4300.8 ± 340.5	4710.1 ± 146.0	4730.2 ± 209.1	0.371	0.365	0.226
TM/g	7211.9 ± 500.6	7220.3 ± 497.0	7744.8 ± 345.1	7725.2 ± 347.8	0.847	0.635	0.412
BF/%	38.4 ± 2.7	40.3 ± 2.6	38.7 ± 2.5	38.2 ± 3.4	0.394	0.827	0.161

Values expressed as the mean ± SEM; *n* = 6; values in a row with superscripts without a common letter differ; *p* < 0.05, Repeated measures ANOVA with Bonferroni post-hoc test. T × D = time × diet interaction; BW = body weight; DEXA = dual energy X-ray absorptiometry; BMC = bone mineral content; BMD = bone mineral density; FM = fat mass; LBM = lean body mass; TM = total mass; and BF = body fat percentage.

**Table 3:** Serum lipid profiles (mmol/L) in 12 chronically obese cats at baseline and after feeding a control food (458.7 mg choline/100 g DM) (n = 6) or a high-choline food (1895.7 mg choline/100g DM) (n = 6) for 5 weeks.

	Control Diet		High Choline Diet		*p*-Value		
	Baseline	5 Weeks	Baseline	5 Weeks	Time	Diet	T × D
CHOL	5.64 ± 0.384	5.61 ± 0.296	4.92 ± 0.210 ^a^	5.98 ± 0.200 ^b^	0.017	0.634	0.013
TAG	0.533 ± 0.033	0.500 ± 0.026	0.483 ± 0.070 ^a^	0.583 ± 0.054 ^b^	0.260	0.799	0.038
HDL-C	5.02 ± 0.304	4.93 ± 0.246	4.51 ± 0.132 ^a^	5.21 ± 0.164 ^b^	0.077	0.679	0.029
NEFA	0.417 ± 0.034	0.515 ± 0.027	0.417 ± 0.046	0.4133 ± 0.038	0.110	0.279	0.090
LDL-C	0.378 ± 0.106	0.453 ± 0.101	0.194 ± 0.107 ^a^	0.503 ± 0.144 ^b^	0.001	0.683	0.014
VLDL-C	0.242 ± 0.015	0.227 ± 0.012	0.220 ± 0.032 ^a^	0.265 ± 0.025 ^b^	0.260	0.799	0.038

Values expressed as the mean ± SEM; *n* = 6; values in a row with superscripts without a common letter differ; *p* < 0.05, Repeated measures ANOVA with Bonferroni post-hoc test. T × D = time × diet interaction; CHOL = cholesterol; TAG = triacylglycerides; HDL-C = high-density lipoprotein cholesterol; NEFA = non-esterified fatty acids; LDL-C = low-density lipoprotein cholesterol; and VLDL = very low-density lipoprotein cholesterol.

## Data Availability

The dataset generated and analysed during the current project is available at the Scholars Portal Dataverse server (https://dataverse.scholarsportal.info/dataset.xhtml?persistentId=doi:10.5683/SP2/SC3TC4, accessed on 24 July 2021).

## References

[1] Scarlett J.M., Donoghue S., Saidla J., Wills J. (1994). Overweight Cats: Prevalence and Risk Factors. Int. J. Obes. Relat. Metab. Disord..

[2] Robertson I.D. (1999). The Influence of Diet and Other Factors on Owner-Perceived Obesity in Privately Owned Cats from Metropolitan Perth, Western Australia. Prev. Vet. Med..

[3] Öhlund M., Palmgren M., Holst B.S. (2018). Overweight in Adult Cats: A Cross-Sectional Study. Acta Vet. Scand..

[4] Teng K.T., McGreevy P.D., Toribio J.A.L.M.L., Raubenheimer D., Kendall K., Dhand N.K. (2017). Risk Factors for Underweight and Overweight in Cats in Metropolitan Sydney, Australia. Prev. Vet. Med..

[5] Vandendriessche V.L., Picavet P., Hesta M. (2017). First Detailed Nutritional Survey in a Referral Companion Animal Population. J. Anim. Physiol. Anim. Nutr..

[6] Diez M., Picavet P., Ricci R., Dequenne M., Renard M., Bongartz A., Farnir F., Diez M., Picavet P., Renard M. (2015). Health Screening to Identify Opportunities to Improve Preventive Medicine in Cats and Dogs. J. Small Anim. Pract..

[7] Cave N.J., Allan F.J., Schokkenbroek S.L., Metekohy C.A.M., Pfeiffer D.U. (2012). A Cross-Sectional Study to Compare Changes in the Prevalence and Risk Factors for Feline Obesity between 1993 and 2007 in New Zealand. Prev. Vet. Med..

[8] Courcier E.A., O’Higgins R., Mellor D.J., Yam P.S. (2010). Prevalence and Risk Factors for Feline Obesity in a First Opinion Practice in Glasgow, Scotland. J. Feline Med. Surg..

[9] Courcier E.A., Mellor D.J., Pendlebury E., Evans C., Yam P.S. (2012). An Investigation into the Epidemiology of Feline Obesity in Great Britain: Results of a Cross-Sectional Study of 47 Companion Animal Practises. Vet. Rec..

[10] Lund E., Armstrong P. (2005). Prevalence and Risk Factors for Obesity in Adult Cats from Private US Veterinary Practices. Int. J. Appl. Res. Vet. Med..

[11] Colliard L., Paragon B.M., Lemuet B., Bénet J.J., Blanchard G. (2009). Prevalence and Risk Factors of Obesity in an Urban Population of Healthy Cats. J. Feline Med. Surg..

[12] Teng K.T., McGreevy P.D., Toribio J.A.L.M.L., Raubenheimer D., Kendall K., Dhand N.K. (2018). Associations of Body Condition Score with Health Conditions Related to Overweight and Obesity in Cats. J. Small Anim. Pract..

[13] Christmann U., Bečvářová I., Werre S.R., Meyer H.P. (2016). Effectiveness of a New Dietetic Weight Management Food to Achieve Weight Loss in Client-Owned Obese Cats. J. Feline Med. Surg..

[14] Hoenig M., Ferguson D.C. (2002). Effects of Neutering on Hormonal Concentrations and Energy Requirements in Male and Female Cats. Am. J. Vet. Res..

[15] Havel P.J., Ramsey J.J., Graham J.L., Kim K., Wei A., Lee A., Fascetti A.J. (2014). Early Effects of Neutering on Energy Expenditure in Adult Male Cats. PLoS ONE.

[16] Villaverde C., Ramsey J.J., Green A.S., Asami D.K., Yoo S., Fascetti A.J. (2018). Energy Restriction Results in a Mass-Adjusted Decrease in Energy Expenditure in Cats That Is Maintained after Weight Regain. J. Nutr..

[17] Deagle G., Holden S.L., Biourge V., Morris P.J., German A.J. (2014). Long-Term Follow-up after Weight Management in Obese Cats. J. Nutr. Sci..

[18] Biourge V.C., Groff J.M., Munn R.J., Kirk C.A., Nyland T.G., Madeiros V.A., Morris J.G., Rogers Q.R. (1994). Experimental Induction of Hepatic Lipidosis in Cats. Am. J. Vet. Res..

[19] National Research Council (2006). Nutrient Requirements of Dogs and Cats.

[20] Brooks D., Churchill J., Fein K., Linder D., Michel K.E., Tudor K., Ward E., Witzel A. (2013). 2014 AAHA Weight Management Guidelines for Dogs and Cats. J. Am. Anim. Hosp. Assoc..

[21] Armstrong P.J., Blanchard G. (2009). Hepatic Lipidosis in Cats. Vet. Clin. N. Am. Small.

[22] Center S.A., Crawford M.A., Guida L., Erb H.N., King J. (1993). A Retrospective Study of 77 Cats With Severe Hepatic Lipidosis: 1975-1990. J. Vet. Intern. Med..

[23] Valtolina C., Favier R.P. (2017). Feline Hepatic Lipidosis. Vet. Clin. N. Am. Small.

[24] Gagne J.M., Weiss D.J., Armstrong P.J. (1996). Histopathologic Evaluation of Feline Inflammatory Liver Disease. Vet. Pathol..

[25] Dimski D., Buffington C., Johnson S., Sherding R., Rosol T. (1992). Serum Lipoprotein Concentrations and Hepatic Lesions in Obese Cats Undergoing Weight Loss. Am. J. Vet. Res..

[26] Verbrugghe A., Bakovic M. (2013). Peculiarities of One-Carbon Metabolism in the Strict Carnivorous Cat and the Role in Feline Hepatic Lipidosis. Nutrients.

[27] Griffin B. (2000). Feline Hepatic Lipidosis: Pathophysiology, Clinical Signs, and Diagnosis. Compend. Contin. Educ. Pract. Vet..

[28] Kuzi S., Segev G., Kedar S., Yas E., Aroch I. (2017). Prognostic Markers in Feline Hepatic Lipidosis: A Retrospective Study of 71 Cats. Vet. Rec..

[29] Zeisel S.H., Da Costa K.A., Franklin P.D., Alexander E.A., Lamont T.A., Sheard N.F., Beiser A. (1991). Choline, an Essential Nutrient for Humans. FASEB J..

[30] Canty D.J., Zeisel S.H. (1994). Lecithin and Choline in Human Health and Disease. Nutr. Rev..

[31] Wang Y.Z., Xu Z.R., Feng J. (2004). The Effect of Betaine and DL-Methionine on Growth Performance and Carcass Characteristics in Meat Ducks. Anim. Feed Sci. Tech..

[32] Esteve-Garcia E., Mack S., de Freitas A.R. (2005). The Effect of DL-Methionine and Betaine on Growth Performance and Carcass Characteristics in Broilers. Anim. Feed Sci. Tech..

[33] Zhan X.A., Li J.X., Xu Z.R., Zhao R.Q. (2006). Effects of Methionine and Betaine Supplementation on Growth Performance, Carcase Composition and Metabolism of Lipids in Male Broilers. Brit. Poult. Sci..

[34] Fernández C., López-Saez A., Gallego L., De La Fuente J.M. (2000). Effect of Source of Betaine on Growth Performance and Carcass Traits in Lambs. Anim. Feed Sci. Tech..

[35] Lawrence B.V., Schinckel A.P., Adeola O., Cera K., Science A. (2002). Impact of Betaine on Pig Finishing Performance and Carcass Composition. J. Anim. Sci..

[36] McDevitt R.M., Mack S., Wallis I.R. (2000). Can Betaine Partially Replace or Enhance the Effect of Methionine by Improving Broiler Growth and Carcase Characteristics?. Brit. Poult. Sci..

[37] Yu D.Y., Xu Z.R., Li W.F. (2004). Effects of Betaine on Growth Performance and Carcass Characteristics in Growing Pigs. Asian Austral. J. Anim..

[38] Schenkel L.C., Sivanesan S., Zhang J., Wuyts B., Taylor A., Verbrugghe A., Bakovic M. (2015). Choline Supplementation Restores Substrate Balance and Alleviates Complications of Pcyt2 Deficiency. J. Nutr. Biochem..

[39] Yao Z., Vance D.E. (1988). The Active Synthesis of Phosphatidylcholine Is Required for Very Low Density Lipoprotein Secretion from Rat Hepatocytes. J. Biol. Chem..

[40] Finkelstein J. (1990). Methionine Metabolism in Mammals. J. Nutr. Biochem..

[41] Vance D.E., Ridgway N.D. (1988). The Methylation of Phosphatidylethanolamine. Prog. Lipid Res..

[42] Rebouche C.J., Seim H. (2002). Carnitine Metabolism and Its Regulation in Microorganisms and Mammals. Annu. Rev. Nutr..

[43] Laflamme D. (1997). Development and Validation of a Body Condition Score System for Cats: A Clinical Tool. Feline Pract..

[44] Horwitz W., Chichilo P., Reynolds H. (1970). Official Methods of Analysis of the Association of Official Analytical Chemists.

[45] Firestone D., American Oil Chemists’ Society (1997). Official Methods and Recommended Practices of the American Oil Chemists’ Society.

[46] Friedewald W.T., Levy R.I., Fredrickson D.S. (1972). Estimation of the Concentration of Low-Density Lipoprotein Cholesterol in Plasma, Without Use of the Preparative Ultracentrifuge. Clin. Chem..

[47] Osorio J.H., Cañas E.Z., Pérez J.E. (2012). Comparison of Lipid Profile in Domestic Cat by Gender and Age. Bol. Cient. Cent. Mus..

[48] De Freitas V.D., Castilho A.R., da Conceição L.A.V., Sousa V.R.F., Mendonça A.J., da Silva F.G., Almeida A.d.B.P.F. (2018). Metabolic Evaluation in Overweight and Obese Cats and Association with Blood Pressure. Cienc. Rural.

[49] Strage E.M., Holst B.S., Nilsson G., Jones B., Lilliehöök I. (2012). Validation of an Enzyme-Linked Immunosorbent Assay for Measurement of Feline Serum Insulin. Vet. Clin. Path..

[50] Appleton D.J., Rand J.S., Sunvold G.D. (2005). Basal Plasma Insulin and Homeostasis Model Assessment (HOMA) Are Indicators of Insulin Sensitivity in Cats. J. Feline Med. Surg..

[51] Appleton D.J., Rand J.S., Sunvold G.D. (2000). Plasma Leptin Concentrations in Cats: Reference Range, Effect of Weight Gain and Relationship with Adiposity as Measured by Dual Energy X-Ray Absorptiometry. J. Feline Med. Surg..

[52] Rizzo C., Boenzi S., Wanders R.J., Duran M., Caruso U., Dionisi-Vici C. (2003). Characteristic Acylcarnitine Profiles in Inherited Defects of Peroxisome Biogenesis: A Novel Tool for Screening Diagnosis Using Tandem Mass Spectrometry. Pediatr. Res..

[53] Vreken P., Van Lint A.E.M., Bootsma A.H., Overmars H., Wanders R.J.A., Van Gennip A.H. (2002). Rapid diagnosis of organic acidemias and fatty-acid oxidation defects by quantitative electrospray tandem-MS acyl-carnitine analysis in plasma. Current Views of Fatty Acid Oxidation and Ketogenesis.

[54] Bjørnvad C.R., Nielsen M.E., Hansen S.E.M., Nielsen D.H. (2017). The Effect of Position on the Precision of Dual-Energy X-Ray Absorptiometry and Correlation with Body Condition Score in Dogs and Cats. J. Nutr. Sci..

[55] Zeisel S.H., Blusztajn J.K. (1994). Choline and Human Nutrition. Annu. Rev. Nutr..

[56] Jiang X., Yan J., Caudill M.A., Zempleni J., Suttie J.W., Gregory J.F., Stover P.J. (2013). Choline. Handbook of Vitamins.

[57] Zeisel S.H. (1990). Choline Deficiency. J. Nutr. Biochem..

[58] Biourge V., Pion P., Lewis J., Morris J.G., Rogers Q.R. (1991). Dietary Management of Idiopathic Feline Hepatic Lipidosis with a Liquid Diet Supplemented with Citrulline and Choline. J. Nutr..

[59] Li Z., Vance D.E.D.E. (2008). Phosphatidylcholine and Choline Homeostasis. J. Lipid. Res..

[60] Yao Z., Vance D.E. (1990). Reduction in VLDL, but Not HDL, in Plasma of Rats Deficient in Choline. Biochem. Cell Biol..

[61] Lombardi B., Pani P., Schlunk F.F. (1968). Choline-Deficiency Fatty Liver: Impaired Release of Hepatic Triglycerides. J. Lipid Res..

[62] Hoffbauer F.W., Zaki F.G. (1963). Fatty Liver due to Choline-Deficiency in the Primate. Topical Problems in Diseases of the Liver.

[63] Handler P., Bernheim F. (1949). Choline Deficiency in the Hamster. Proc. Soc. Exp. Biol. Med..

[64] Chahl J.S., Kratzing C.C. (1973). Fatty Acid Composition of Tissue Lipids in Choline Deficient Rats. Q. J. Exp. Physiol. Cogn. Med. Sci..

[65] Clark M.H., Larsen R., Lu W., Hoenig M. (2013). Investigation of 1H MRS for Quantification of Hepatic Triglyceride in Lean and Obese Cats. Res. Vet. Sci..

[66] Cole L.K., Vance J.E., Vance D.E. (2012). Phosphatidylcholine Biosynthesis and Lipoprotein Metabolism. Biochim. Biophys. Acta Mol. Cell Biol. Lipids.

[67] Fielding P.E., Fielding C.J. (2002). Dynamics of lipoprotein transport in the human circulatory system. New Comprehensive Biochemistry.

[68] Jordan E., Kley S., Le N.-A., Waldron M., Hoenig M. (2008). Dyslipidemia in Obese Cats. Domest. Anim. Endocrinol..

[69] Bauer J.E. (2004). Lipoprotein-Mediated Transport of Dietary and Synthesized Lipids and Lipid Abnormalities of Dogs and Cats. JAVMA J. Am. Vet. Med. Assocc..

[70] Russell J.C., Proctor S.D. (2006). Small Animal Models of Cardiovascular Disease: Tools for the Study of the Roles of Metabolic Syndrome, Dyslipidemia, and Atherosclerosis. Cardiovasc. Pathol..

[71] Tinoco J., Shannon A., Lyman R.L. (1964). Serum Lipids in Choline-Deficient Male and Female Rats. J. Lipid Res..

[72] Wilgram G.F., Lewis L.A., Best C.H. (1957). Effect of Choline and Cholesterol on Lipoprotein Patterns of Rats. Circ. Res..

[73] Li H., Wang H., Yu L., Wang M., Liu S., Sun L., Chen Q. (2015). Effects of Supplementation of Rumen-Protected Choline on Growth Performance, Meat Quality and Gene Expression in Longissimus Dorsi Muscle of Lambs. Null.

[74] Lien T.F., Jan D.F. (1999). The Effect on the Lipid Metabolism of Tsaiya Ducks When High Levels of Choline or Methionine Are Added to the Ducks’ Diet. Asian Austral. J. Anim. Sci..

[75] Buchman A.L., Ament M.E., Sohel M., Dubin M., Jenden D.J., Roch M., Pownall H., Farley W., Awal M., Ahn C. (2001). Choline Deficiency Causes Reversible Hepatic Abnormalities in Patients Receiving Parenteral Nutrition: Proof of a Human Choline Requirement: A Placebo-controlled Trial. J. Parenter. Enter. Nutr..

[76] Olthof M.R., Brink E.J., Katan M.B., Verhoef P. (2005). Choline Supplemented as Phosphatidylcholine Decreases Fasting and Postmethionine-Loading Plasma Homocysteine Concentrations in Healthy Men. Am. J. Clin. Nutr..

[77] Rahmani M.G., Kamalyan R.G., Dehghan-Banadaky M.J., Marmaryan G.Y. (2012). The Effect of Oral Administration of Choline on Some Liver Function Characterized Blood Plasma Enzymes of Early Lactating Dairy Cows. Biol. J. Armen..

[78] Getty C.M., Dilger R.N. (2015). Moderate Perinatal Choline Deficiency Elicits Altered Physiology and Metabolomic Profiles in the Piglet. PLoS ONE.

[79] Center S.A. (2005). Feline Hepatic Lipidosis. Vet. Clin. N. Am. Small.

[80] Everett R.M., Duncan J.R., Prasse K.W. (1977). Alkaline Phosphatase, Leucine Aminopeptidase, and Alanine Aminotransferase Activities with Obstructive and Toxic Hepatic Disease in Cats. Am. J. Vet. Res..

[81] Holm P.I., Hustad S., Ueland P.M., Vollset S.E., Grotmol T., Schneede J. (2007). Modulation of the Homocysteine-Betaine Relationship by Methylenetetrahydrofolate Reductase 677 C->T Genotypes and B-Vitamin Status in a Large-Scale Epidemiological Study. J. Clin. Endocrinol. Metab..

[82] Lin C.S., Wu R.D. (1986). Choline Oxidation and Choline Dehydrogenase. J. Protein Chem..

[83] Ueland P.M., Holm P.I., Hustad S. (2005). Betaine: A Key Modulator of One-Carbon Metabolism and Homocysteine Status. Clin. Chem. Lab. Med..

[84] Kohlmeier M., Kohlmeier M. (2015). Chapter 8—Amino Acids and Nitrogen Compounds. Nutrient Metabolism.

[85] Barak A., Beckenhauer H., Uma D. (1996). Betaine, Ethanol, and the Liver: A Review. Alcohol.

[86] Barak A.J., Beckenhauer H.C., Tuma D.J. (1982). Use of S-Adenosylmethionine as an Index of Methionine Recycling in Rat Liver Slices. Anal. Biochem..

[87] Poirier L.A., Grantham P.H., Rogers A.E. (1977). The Effects of a Marginally Lipotrope-Deficient Diet on the Hepatic Levels of S-Adenosylmethionine and on the Urinary Metabolites of 2-Acetylaminofluorene in Rats. Cancer Res..

[88] Shivapurkar N., Poirier L.A. (1983). Tissue Levels of S-Adenosylmethionine and S-Adenosylhomocysteine in Rats Fed Methyl-Deficient, Amino Acid-Defined Diets for One to Five Weeks. Carcinogenesis.

[89] Zeisel S.H., Zola T., da Costa K.-A., Pomfret E.A. (1989). Effect of Choline Deficiency on S-Adenosylmethionine and Methionine Concentrations in Rat Liver. Biochem. J..

[90] Stead L.M., Brosnan J.T., Brosnan M.E., Vance D.E., Jacobs R.L. (2006). Is It Time to Reevaluate Methyl Balance in Humans?. Am. J. Clin. Nutr..

[91] Wei Y., Rector R., Thyfault J., Ibdath J. (2008). Non-Alcoholic Fatty Liver Disease and Mitochondrial Dysfunction. World J. Gastroenterol..

[92] Mihalik S.J., Goodpaster B.H., Kelley D.E., Chace D.H., Vockley J., Toledo F.G.S., DeLany J.P. (2010). Increased Levels of Plasma Acylcarnitines in Obesity and Type 2 Diabetes and Identification of a Marker of Glucolipotoxicity. Obesity.

[93] Dahlhoff C., Worsch S., Sailer M., Hummel B.A., Fiamoncini J., Uebel K., Obeid R., Scherling C., Geisel J., Bader B.L. (2014). Methyl-Donor Supplementation in Obese Mice Prevents the Progression of NAFLD, Activates AMPK and Decreases Acyl-Carnitine Levels. Mol. Metab..

[94] Hoppel C.L., Genuth S.M. (1980). Carnitine Metabolism in Normal-Weight and Obese Human Subjects during Fasting. Am. J. Physiol. Endocinol. Metab..

[95] Koves T.R., Li P., An J., Akimoto T., Slentz D., Ilkayeva O., Dohm G.L., Yan Z., Newgard C.B., Muoio D.M. (2005). Peroxisome Proliferator-Activated Receptor-γ Co-Activator 1α-Mediated Metabolic Remodeling of Skeletal Myocytes Mimics Exercise Training and Reverses Lipid-Induced Mitochondrial Inefficiency. J. Biol. Chem..

[96] Newgard C.B., An J., Bain J.R., Muehlbauer M.J., Stevens R.D., Lien L.F., Haqq A.M., Shah S.H., Arlotto M., Slentz C.A. (2009). A Branched-Chain Amino Acid-Related Metabolic Signature That Differentiates Obese and Lean Humans and Contributes to Insulin Resistance. Cell Metab..

[97] Sivanesan S., Taylor A., Zhang J., Bakovic M. (2018). Betaine and Choline Improve Lipid Homeostasis in Obesity by Participation in Mitochondrial Oxidative Demethylation. Front. Nutr..

[98] Kalhan S.C., Guo L., Edmison J., Dasarathy S., McCullough A.J., Hanson R.W., Milburn M. (2011). Plasma Metabolomic Profile in Nonalcoholic Fatty Liver Disease. Metabolism.

[99] Sampey B.P., Freemerman A.J., Zhang J., Kuan P.-F., Galanko J.A., O’Connell T.M., Ilkayeva O.R., Muehlbauer M.J., Stevens R.D., Newgard C.B. (2012). Metabolomic Profiling Reveals Mitochondrial-Derived Lipid Biomarkers That Drive Obesity-Associated Inflammation. PLoS ONE.

[100] Buchman A.L., Dubin M.D., Moukarzel A.A., Jenden D.J., Roch M., Rice K.M., Gornbein J., Ament M.E. (1995). Choline Deficiency: A Cause of Hepatic Steatosis during Parenteral Nutrition That Can Be Reversed with Intravenous Choline Supplementation. Hepatology.

[101] Spencer M.D., Hamp T.J., Reid R.W., Fischer L.M., Zeisel S.H., Fodor A.A. (2011). Association between Composition of the Human Gastrointestinal Microbiome and Development of Fatty Liver with Choline Deficiency. Gastroenterology.

[102] Michel V., Singh R.K., Bakovic M. (2011). The Impact of Choline Availability on Muscle Lipid Metabolism. Food Funct..

[103] Schaeffer M.C., Rogers Q.R., Morris J.G. (1982). The Choline Requirement of the Growing Kitten in the Presence of Just Adequate Dietary Methionine. Nutr. Res..

[104] Grant C.E., Chan J., Shoveller A.K., Bakovic M., Blois S., Fascetti A.J., Yu J.Z., Verbrugghe A. (2021). Theoretical Intake of Amino Acids and Vitamins in Obese Cats Undergoing Energy Restriction Using Veterinary Therapeutic Diets for Weight Loss Compared to over the Counter Diets. BMC Vet. Res..

[105] Grant C.E., Shoveller A.K., Blois S., Bakovic M., Monteith G., Verbrugghe A. (2020). Dietary Intake of Amino Acids and Vitamins Compared to NRC Requirements in Obese Cats Undergoing Energy Restriction for Weight Loss. BMC Vet. Res..

[106] Wilson S.A., Villaverde C., Fascetti A.J., Larsen J.A. (2019). Evaluation of the Nutritional Adequacy of Recipes for Home-Prepared Maintenance Diets for Cats. JAVMA J. Am. Vet. Med. Assocc..

[107] Blanchard G., Paragon B.M., Serougne C., Lutton C., Ferezou J., Milliat F., Lutton C. (2004). Plasma Lipids, Lipoprotein Composition and Profile during Induction and Treatment of Hepatic Lipidosis in Cats and the Metabolic Effect of One Daily Meal in Healthy Cats. J. Anim. Physiol. Anim. Nutr..

